# BDA: A novel method for identifying defects in body-centered cubic crystals

**DOI:** 10.1016/j.mex.2016.03.013

**Published:** 2016-03-31

**Authors:** Johannes J. Möller, Erik Bitzek

**Affiliations:** Friedrich-Alexander-Universität Erlangen-Nürnberg, Department of Materials Science and Engineering, Institute I, Martensstr. 5, D-91058 Erlangen, Germany

**Keywords:** Atomistic simulations, Molecular dynamics, Defect analysis, bcc materials

## Abstract

The accurate and fast identification of crystallographic defects plays a key role for the analysis of atomistic simulation output data. For face-centered cubic (fcc) metals, most existing structure analysis tools allow for the direct distinction of common defects, such as stacking faults or certain low-index surfaces. For body-centered cubic (bcc) metals, on the other hand, a robust way to identify such defects is currently not easily available.

We therefore introduce a new method for analyzing atomistic configurations of bcc metals, the BCC Defect Analysis (BDA). It uses existing structure analysis algorithms and combines their results to uniquely distinguish between typical defects in bcc metals.

In essence, the BDA method offers the following features:

• Identification of typical defect structures in bcc metals.
• Reduction of erroneously identified defects by iterative comparison to the defects in the atom's neighborhood.
• Availability as ready-to-use Python script for the widespread visualization tool OVITO [http://ovito.org].

## Graphical abstract

**Figure:**
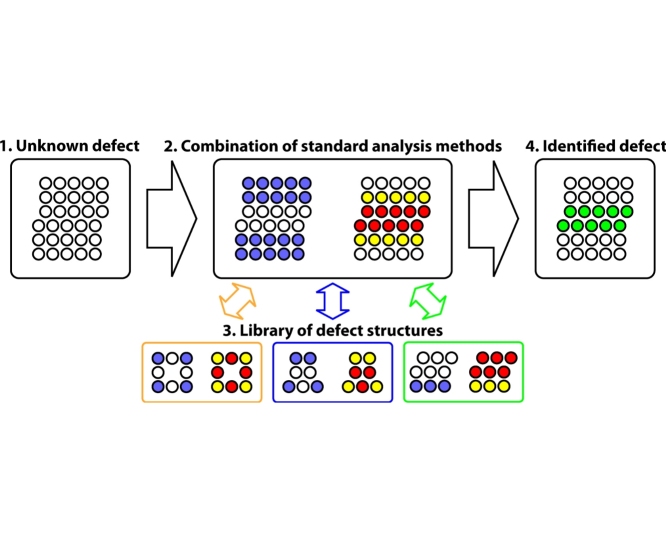

## Method

The interpretation and analysis of atomistic simulations of condensed matter hinges on the automatized identification of (lattice) defects [1], [2], which is therefore a key element of many tools for atomic structure visualization [3], [4], [5]. While many different crystallographic parameters and algorithms have been developed for face-centered cubic (fcc) crystals [4], [6], few methods are specifically adapted to the identification of defects in body centered cubic (bcc) crystals. An unambiguous, automatized classification of bcc crystal defects is of particular importance as large-scale atomistic simulations are increasingly used to study specific aspects of bcc plasticity and failure [7], [8], [9], [10], including nanocrystal plasticity [11], dislocation-defect interactions [12], [13], [14], fracture [15], and in particular irradiation damage [16].

Here, we introduce a new method for analyzing atomistic configurations of bcc metals, the BCC Defect Analysis (BDA). It uses the results of the widely-used coordination number (CN), centrosymmetry parameter (CSP) [17], and common neighbor analysis (CNA) [18] to identify defects that are typical for bcc crystals. The first step within the BDA approach is to analyze all atoms in a given configuration with the CN, CSP, and CNA techniques. Here, it is noteworthy that the cutoff radius for the CN is $r_{c}=(1+\sqrt{2})/2a_{0}$ with *a*_0_ being the lattice parameter. Since the six next-nearest neighbors of perfect bcc atoms are within this cutoff distance, their CN increases from 8 to 14. Then, the CN and CSP values of each atom, which is not in a bcc environment according to the CNA or has a CN of 14, are compared to empirically determined values for the following typical defects: surfaces, vacancies, twin boundaries, screw dislocations, {110} planar faults, and edge dislocations. The criteria for this pre-characterization are presented in the first three columns of Table 1.

The novelty of the BDA method is that not only the atom itself, but also each of its neighbors is evaluated against characteristic defect criteria. To this end, all atoms within the cutoff distance are classified according to their values of CN and CSP. *N*_p_ denotes the number of neighbor atoms being in a perfect bcc environment. The non-perfect neighbor atoms are compared to a number of different criteria, see the column nos. 5–9 in Table 1. If a neighbor atom fulfills a criterion, i.e., a certain combination of CN and CSP, the number *N*_d_ is increased for the respective criterion. If the occurrences of *N*_p_ and *N*_d_ match the characteristic occurrences for a defect, this defect is assigned to the atom. If not, the atom and its neighbors are evaluated against the criteria for the next defect in Table 1. This comparison is performed for all non-bcc atoms in the configuration. To minimize the number of erroneously identified defects, every identified defect is then compared to its neighboring defects and is flagged as ‘unidentified’ if it is not representing the relative majority among its neighbors. In the final step, all unidentified atoms are assigned to the predominant defect in their neighborhood. As a result, the number of unidentified defects is reduced by repeating this comparison until the number of unidentified defects is smaller than a threshold value or does not change upon further repetition. This step is optional, but recommended, since it homogenizes the resulting output data.

In essence, the BDA method consists of the following steps:

1 Calculate a-CNA [2], CSP [17], and CN (with cutoff radius $r_{c}=(1+\sqrt{2})/2a_{0}$ to include also next-nearest neighbors) for all atoms.
2 Generate list of non-bcc neighbors, i.e., with a-CNA ≠ bcc or CN ≠ 14, for each atom.
3 For defects in Table 1: test for the given criteria and proceed with no. 5 in case of positive match. In this context, it is important to test for the different defects in the ordering as they are presented in Table 1.
4 Append atoms, for which no match was found, to the list of unidentified defects.
5 If the identified defect is not representing the relative majority of the defects in its neighborhood (excluding unidentified atoms) or occurs less than two times: append atom to list of unidentified defects.
6 For all atoms in the list of unidentified defects: assign the defect, which represents the relative majority of all neighbors’ defects and occurs more than two times, to the atom; remove the atom from the list of unidentified atoms.
7 Repeat no. 6 until the number of unidentified atoms is smaller than a threshold value or does not change upon further repetition.

The recommended color scheme to visualize the output data of the BDA is given in Table 2. It is based on the ‘cubehelix’ approach [19] and yields images where the different defects can be distinguished even if printed with most B/W printers.

The BDA method is currently implemented as a Python script for OVITO's scripting interface [20] and is available as an open-source tool [21] under the GNU General Public License (GPL) v3.

## Additional information

In this section, we first provide additional information about the existing structure analysis algorithms that were used to develop the BDA method. The details of the evaluated defect structures, which we have used to parameterize our method, are presented thereafter. In the end, we briefly comment on the use of the BDA method at high temperatures, the inclusion of additional defects, and the computational effort of the BDA.

### Existing methods for structure analysis

The following paragraphs briefly summarize the basic ideas of each of the aforementioned existing analysis methods. At the end, the results of the existing techniques are compared to the newly developed BDA method for an application example.

*Energy filter*. Only atoms with increased potential energy (typically *E*_pot_ > 0.98*E*_0_) are displayed. Here, *E*_0_ denotes the cohesive energy per atom, which is typically negative. This filtering method makes use of the fact that atoms in the neighborhood of defects are usually in an higher energy state compared to atoms in a perfect bulk configuration. When applied on-the-fly, i.e., at the run time of the simulation, it can also be used to drastically reduce the simulation output data. This method, however, works well only for simulations at low temperatures.

*Common neighbor analysis (CNA)*. Each bond that connects a central atom and its near neighbors is characterized by a set of four characteristic numbers *ijkl* [18], [2]. These numbers depend on whether the atoms are nearest (1) or next-nearest (2) neighbors (*i*), the number of near neighbors that they have in common (*j*), the number of bonds among these common neighbors (*k*), and the number of bonds in the longest continuous chain of bonds connecting the common neighbors (*l*). It is important to note that atoms are treated as near neighbors, which are consequently connected by bonds, if they are within a certain distance of each other. In fcc structures (characterized by a CNA number of 1421) this distance lies between the first and the second nearest neighbors and thus only bonds between nearest neighbors are counted. For bcc structures, however, also second nearest neighbors are taken into account and the cutoff distance should lie between the second and the third neighbor shell. A bcc environment is then characterized by eight nearest neighbor bonds having a CNA number of 1666 and six next-nearest neighbor bonds with a CNA number of 2444. With the adaptive common neighbor analysis (a-CNA) implemented in OVITO the optimal cutoff radius for each particle is automatically identified [2].

*Coordination number (CN)*. The number of atoms within a cutoff distance is counted. This distance typically lies between the nearest (nn) and second nearest neighbor (nn2) distance to count only nearest neighbor atoms. Using a cutoff radius between the second and third nearest neighbor distance can increase the sensitivity of this characterization method particularly for bcc metals, where the inclusion of second nearest neighbor interactions is important for the correct description of the material's behavior [22]. Atoms that have the coordination of the corresponding perfect host lattice (CN(nn) = 8 or CN(nn2) = 14 for bcc) are usually not shown in the visualization of defects. This method is, however, unable to identify twinned structures or screw dislocation cores, where the number of neighboring atoms does not change compared to the perfect crystal.

*Centrosymmetry parameter (CSP)*. The difference of the vectors pointing to opposite nearest neighbor pairs are summed [17]. In this manner, the local lattice distortion around an atom can be characterized. For a perfect bulk structure all vectors cancel out and the CSP is zero, but whenever the symmetry around an atom is broken, e.g., in presence of a vacancy or a surface, it takes on values larger than zero. A necessary input parameter for this analysis is the number of nearest neighbors in the host lattice. The CSP is a useful characterization method to detect typical defects in the bcc lattice, e.g., twin boundaries, vacancies, and dislocations, but not necessarily to distinguish them uniquely.

**Fig. 1:**
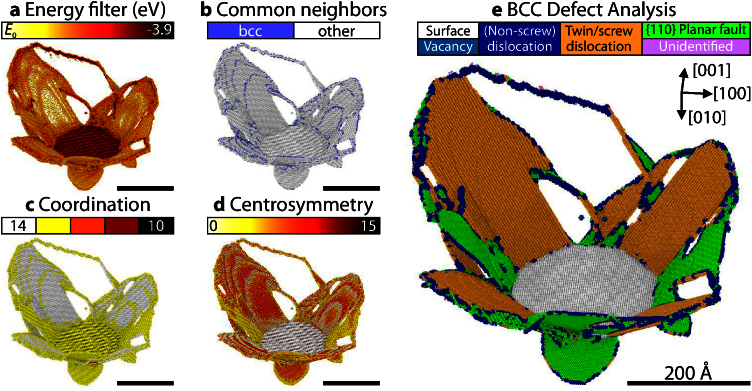
Comparison of different analysis techniques for the plastic region around a penny-shaped crack on the (010) plane in Fe. Atomic interactions: Mendelev-II potential [28]; atoms are only shown in subfigures (b–d), if they are not classified as bcc due to CNA [18]*and* do not exhibit a coordination number of 14. (a) Energy filtered configuration where atoms with a potential energy lower than -4.05 eV are not shown; (b) a-CNA [20], [2]; (c) CN including next-nearest neighbors; (d) CSP [17]; (e) defects according to the newly developed BCC Defect Analysis (BDA); with the BDA, all relevant defects are robustly identified; the other methods (a–d), on the other hand, are not able to uniquely distinguish between all the different defect types.

The significant advantage of the BDA method becomes evident when the different techniques are compared for a configuration that contains a large number of different defects. Such an example is provided in Fig. 1, which shows the complex plastic zone that forms around a penny-shaped crack on the (010) plane in Fe, see Refs. [23], [24] for details. For instance, both edge-oriented dislocations and surfaces fall into the same energy range with the energy filtering method (a). At the same time, atoms in the interior of a twin exhibit the same energy as the perfect structure and the twin therefore appears semi-transparent in the figure. The CNA, on the other hand, reliably detects all defects, but is not able to distinguish between them (b). Using the coordination analysis, the (010) surface and the dislocations appear in the same color (c). Likewise, twin boundaries and {110} planar faults exhibit similar centrosymmetry parameters (d). The new BDA method, however, clearly distinguishes between surfaces, planar faults, twin boundaries, dislocations, and vacancies (d).

### Evaluated defect structures

**Fig. 2:**
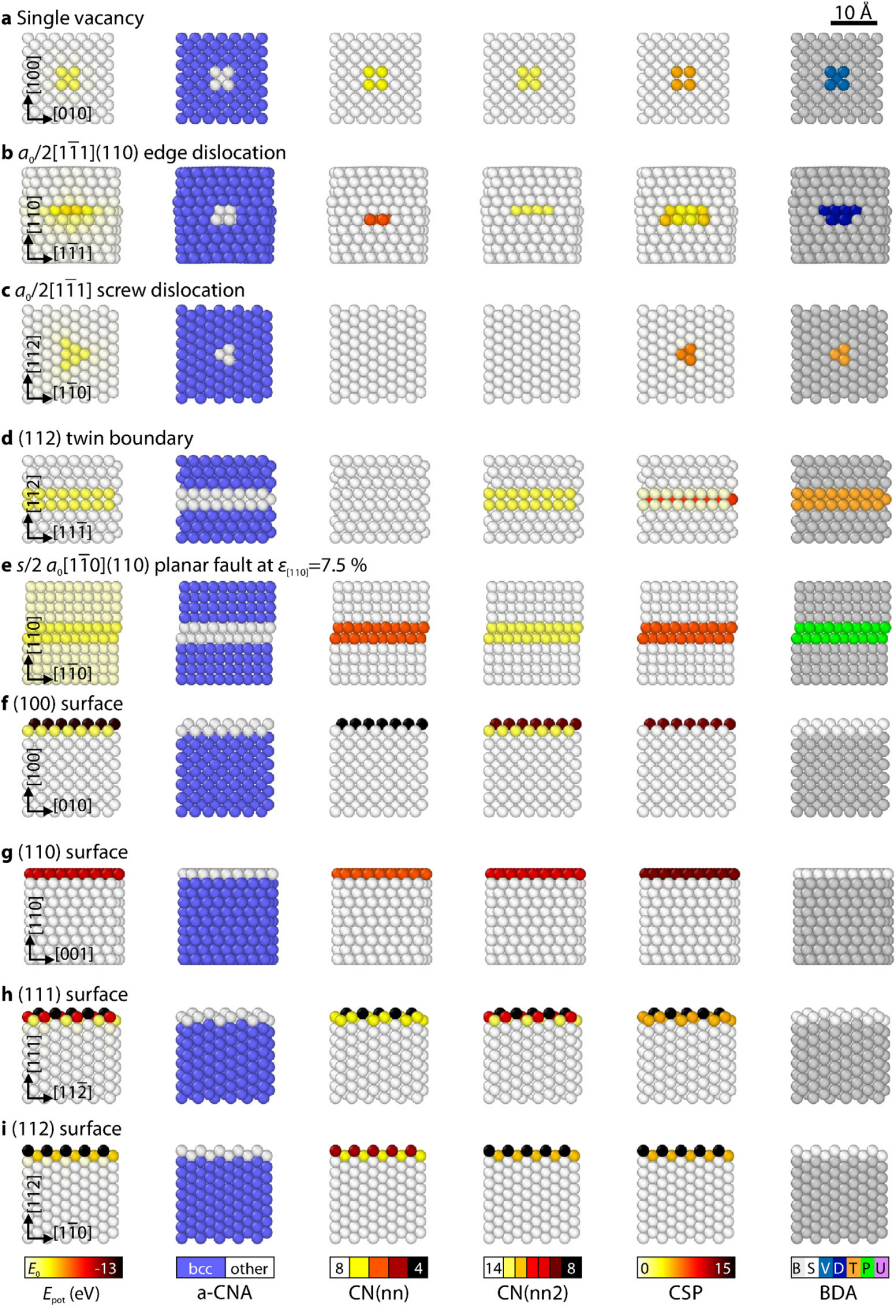
Overview oftypical crystallographic defects in bcc metals as identified by different characterization methods: energy filtering, a-CNA [2], CN including first (nn) and second nearest neighbors (nn2), CSP [17] and the newly developed BCC Defect Analysis (BDA); note the following abbreviations in the color scheme of the BDA: B: bulk, S: surface, V: vacancy; D: (non-screw) dislocation, T: (112) twin boundary or screw dislocation, P: (110) planar fault, U: unidentified; the Chiesa potential for Fe [26] was used here. (For interpretation of the references to color in this figure legend, the reader is referred to the web version of this article.)

To identify the typical values of common bcc defects for both coordination and centrosymmetry, see Table 1, a number of different ideal (in an energetic minimum) and excited systems (during dynamic simulations starting at *T* = 0 K) were evaluated. The configurations contained the following known defects: (100), (110), (111), and (112) surfaces, mono- and di-vacancies as well as vacancy rows, twin boundaries on 112 planes, planar faults on {110} planes, screw dislocations, and edge dislocations on {110} and {112} planes. Fig. 2 gives an overview of most of the different defects as identified with the aforementioned characterization methods and the new BDA method. The figure presents the defects as obtained with the Chiesa potential [25], [26] for Fe. As different potentials can show different defect structures [27], selected simulations were performed and analyzed using the Fe potentials ‘Mendelev-II’ [28] and ‘Chamati’ [29]. The following paragraphs summarize the most important subtleties and issues that occurred during the characterization of the defects.

*Vacancies*. Atoms in the vicinity of a mono-vacancy, see Fig. 2a, are missing one neighbor and thus exhibit a coordination of 13. Such an under-coordinated state, as we will see later, is, however, also common to most other defects. For this reason, the centrosymmetry parameter is used here as additional indicator. Nearest neighbors to a vacancy exhibit a relatively high CSP, for which a value of 4 was found as a good lower limit. Next-nearest neighbors, which are also 13-coordinated, have comparably low CSP values lower than unity. For di-vacancies and vacancy rows, the characterization has to additionally account for 12-coordinated atoms, but the CSP thresholds of mono-vacancies are still valid.

*Edge dislocations*. Unlike near vacancies, atoms in the vicinity of edge dislocations exhibit less well-defined characteristics. Especially under dynamic and 3D conditions, the dislocation structure is not as simple as the static structure of a pure edge dislocation shown in Fig. 2b. When the dislocation moves, for instance, it is spread out over some atomic distances on its glide plane and a variety of coordination numbers are frequently observed ranging from 12 to up to 15. Further characterization according to the CSP was difficult as the determined CSP values were highly depended on the used interatomic potential. There is, however, a high possibility for the presence of a (non-screw) dislocation if the number of non-14-coordinated neighbors is higher than the number of 14-coordinated neighbors. Since this criterion is relatively weak, it is important to note, that the (non-screw) dislocation is the *last* defect type, which is tested in the BDA approach, see Table 1. That means, if all other criteria do not apply, the given defect structure can only be a (non-screw) dislocation or an unknown defect. As a consequence, the tips of twins and planar faults are frequently identified as (non-screw) dislocations, see Fig. 1e. Since these tips generally consist of partial dislocations, the identification as ‘dislocation’ is of course not wrong per se. It has to be noted in this context, that some tilt grain boundaries in Fe and W exhibit the same characteristics as (non-screw) dislocations. This observation is interesting since the relative tilt rotation of two grains can be always expressed by the accumulation of many dislocations [30]. Both defect types can, however, still be differentiated since grain boundaries are planar and dislocations are linear defects. This double identification does therefore not limit the applicability of the BDA method. This current limitation of the BDA method can be overcome by using more advanced and computationally more expensive methods, such as the *Crystal Analysis Tool* [31].

*Screw dislocations*. Screw dislocations are treated separately from edge dislocations for two reasons: first, screw dislocations can easily cross slip and therefore have a generally different glide behavior as compared to edge dislocations. As a result, it is appealing to directly find them in a given configuration. Second, their line direction corresponds to the shearing direction to produce a deformation twin. For this reason, it was not possible to distinguish their characteristics from those of twin boundaries. Dislocations are, however, line defects and can thus easily be differentiated from the planar twin structures by comparing the defects’ dimensionalities. As this comparison is not (yet) implemented in the BDA algorithm, the precise defect identification has to be done by visual inspection of the analysis output in this case. Unlike around edge dislocations, atoms in the neighborhood of screw dislocations are generally not under-coordinated, see Fig. 2c. Instead, they exhibit a coordination of 14 and have a relatively high number of 14-coordinated neighbors, which are not identified as bcc by the CNA.

*Twin boundaries*. Twin boundaries contain both 13- and 14-coordinated atoms, see Fig. 2d. Two main issues, however, significantly complicate their further characterization: first, the precise twin boundary structure determined with different interatomic potentials frequently differs from the ideal one shown in the figure. Second, in dynamically evolving structures *a*_0_/6 〈1 1 1〉 twinning dislocations glide on existing twin boundaries. Twinning dislocations are often detected as bcc by the CNA, see Fig. 1b, and characterized by a comparably high CSP (>8), but less than 9 perfect bcc neighbors. Atoms within the twin boundary close to such a twinning dislocation, are missing one neighbor atom and have either a low CSP (<1) but four 14-coordinated neighbors with CSP>8 or a relatively high CSP (>4.5) and fixed numbers of perfect, 13-, and 14-coordinated neighbors.

{110} *planar faults*. Planar faults of $s/2[1\bar{1}0](110)$ type, where *s* ≈ 1/3, are included in the list of known defects since many EAM potentials for bcc metals predict their formation [27]. Their occurrence is, however, limited to highly stressed areas, e.g., in the close vicinity of a crack tip – even under fully three-dimensional conditions [23]. The planar fault presented in Fig. 2e is therefore shown for an applied tensile strain of *ɛ*_[110]_ = 7.5%. Planar faults are 13-coordinated at the boundary layer between the fault and the perfect lattice (shown in Fig. 2e) and 12-coordinated in the interior of the defected region (not shown in Fig. 2e). Similar to twin boundaries, further complications arise due to the glide of partial dislocations of $s/2[1\bar{1}0]$ type on the existing fault. It should finally be noted, that the existence of planar faults is most probably due to an artificial minimum in the generalized stacking fault energy surface of many EAM potentials under applied uni-axial or equi-biaxial strains [32].

*Surfaces*. The surface structures of (100), (110), (111), and (112) surfaces are shown in Fig. 2f–i. Since the bcc lattice is not a dense-packed structure, even these low-index surface planes do not exhibit unique coordination numbers, as e.g., low-index surfaces up to the (111) plane in the fcc structure. Here, the (110) surface is the only exception, see Fig. 2g, which exhibits a unique CN value of 10. For a correct identification of an atom sitting at a surface, it is sufficient, if the atom's CN is lower than 12 *or* if it has more than three neighbors, which fulfill this criterion, and while being under-coordinated itself.

### BDA at high temperatures

**Fig. 3:**
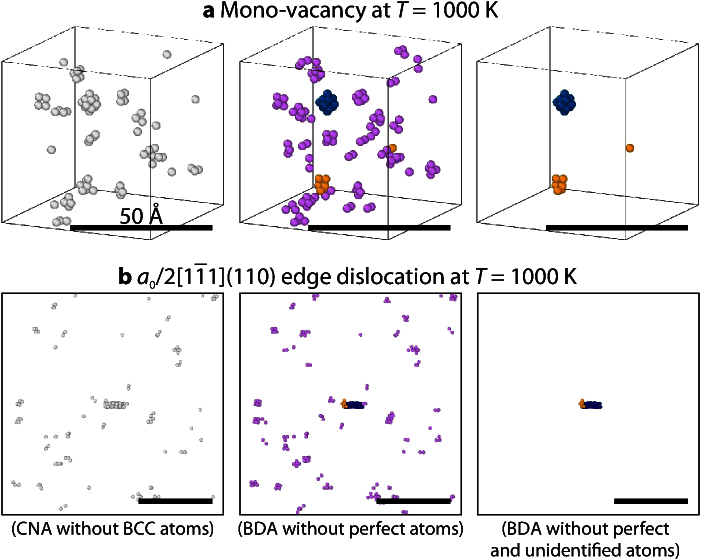
Comparison of the CNA and BDA techniques for defect structures at high-temperature (*T* = 1000 K). (a) Mono-vacancy; (b) edge dislocation; the CNA encounters a multitude of non-bcc atoms, which makes it difficult to separate the thermal noise from the actual defect (left subfigures); the BDA, on the contrary, correctly detects both the vacancy and the edge dislocation even at 1000 K (center subfigures); when unidentified defects are not shown (right subfigures), the BDA can also be used to significantly reduce the thermal noise. Color schemes for CNA and BDA as in Fig. 1.

To demonstrate the applicability of the BDA also at elevated temperatures, NVE simulations of selected defect structures were performed and analyzed for *T* = 1000 K, which is approximately 50% of the melting temperature. Fig. 3 shows the high-temperature configurations of the mono-vacancy and the edge dislocation after *t* = 20 ps of equilibration. The figure further compares the well-established CNA to the newly developed BDA technique. While the CNA detects a multitude of non-bcc atoms, the BDA correctly identifies the vacancy and the edge dislocation even at *T* = 1000 K. Most other non-bcc atoms are not assigned to any other defect type with the exception of spurious atomic clusters that were erroneously identified as screw dislocations or twin boundaries (orange). The zero-dimensionality of these clusters, however, clearly contradicts to such defect types, which have to be either one- or two-dimensional.

At high temperatures, the BDA can even be used to reduce the thermal noise. This is shown in the right subfigures of Fig. 3, where unidentified atoms are not printed. In this manner, those atoms are not visualized, which deviate from the bcc structure only as a result of thermal vibrations. The resulting pictures mainly contain the introduced defects, do not suffer from confusing thermal noise, and may thus help to clearly convey a scientific message.

### Inclusion of additional defects

Although the BDA method has been originally developed for only the defect types presented before, it is not limited to them. This means, that it can be readily extended to include additional defects, such as interstitial atoms, which are particularly important in the context of irradiation damage [7], [33], [34], [12], [35]. For this purpose, the characteristic values of CN and CSP have to be found for a new defect class at first. It must then be carefully examined how the new criteria influence on the already implemented defects. This evaluation finally determines the new ordering, in which the BDA algorithm tests for the different criteria, cf. Table 1. The ordering must not, but can, be crucial for the correct defect identification since a certain defect could in principle fulfill different criteria.

### Computational effort

To estimate the necessary computational effort of the BDA, the configuration shown in Fig. 1 was evaluated. It contained approximately 57.3 million atoms (5.4 GB file size), of which only 4% are defect atoms. The corresponding output file size was only 8.6 MB. With the current OVITO version 2.5.1 [20], the CNA, CN, and CSP needed each approximately 18% of the total computation time. The final BDA run, which only operated on the 4% defect atoms, needed approximately 4% of the total time. The remainder to 100% was spent with I/O and the calculation of the neighbor list for each atom. It is important to note, that the additional time needed for the final optimization run was negligible, but lead to only 0.6% unidentified defects in comparison to all defects in the configuration. Without optimization, 5% of all defect atoms were unidentified. The total time spent for this calculation was approximately 15 min on a workstation with an Intel Xeon Processor E5-2697 v2 (2.7 GHz) and 128 GB RAM.

## Figures and Tables

**Table 1:** Overview of criteria for common bcc defects in the BCC Defect Analysis (BDA). CNA: common neighbor analysis [18] in OVITO's *adaptive* mode implementation (a-CNA) [20], [4], [2]; CN: coordination number for cutoff radius $r_{c}=(1+\sqrt{2})/2a_{0}$; CSP: centrosymmetry parameter [17]; *N*_d_(CN;CSP) is the number of non-bcc (CNA ≠ bcc) neighbors fulfilling the criterion for both CN and CSP; *N*_p_ is the number of neighbors in a perfect bcc environment (CNA = bcc and CN = 14).

Surface (S)				
CNA	CN	CSP	*N*_p_	*N*_d_(<12;–)
≠bcc	<12	–	–	–
≠bcc	<14	–	–	>3

Vacancy and vacancy row (V)								
CNA	CN	CSP	*N*_*p*_	*N*_**d**_(12 ; <1)	*N*_d_(12 ; >4)	*N*_d_(13 ; <1)	*N*_d_(13 ; >4)	*N*_d_(13;–)
≠bcc	=12	<1	6	–	2	–	4	–
≠bcc	=12	>4	3	2	1	2	4	–
≠bcc	=13	<1	9	–	–	–	4	–
				–	2	–	2	–
≠bcc	=13	>4	7	–	–	3	3	–
				2	2	1	1	–
				–	–	–	–	6
			>7	–	–	–	4	–

Twin boundary and screw dislocation (T)						
CNA	CN	CSP	*N*_p_	*N*_d_(13;–)	*N*_d_(14;–)	*N*_d_(14 ; >8)
≠bcc	=13	<1	–	–	–	4
≠bcc	=13	>4.5	6	5	2	–
–	=14	–	[6..9]	–	>3	–
				4	2	–
–	=14	>8	<9	–	–	–

{110} planar fault (P)						
CNA	CN	CSP	*N*_p_	*N*_d_(12;–)	*N*_d_(13;–)	*N*_d_([12 . .13];–)
≠bcc	=12	–	0	>8	–	–
				[3..6]	[7..9]	–
				>5	>2	–
≠bcc	=13	–	–	–	>6	9
			4	3	6	–
			>5	<2	6	–
			<4	<5	>6	–

(Non-screw) dislocation (D)						
CNA	CN	CSP	*N*_p_	*N*_d_(≠14;–)	*N*_d_(14;–)	*N*_d_(>11, ≠ 14;–)
≠bcc	>11,≠14	–	–	>N_d_(14;–)	<N_d_(≠14;)	–
≠bcc	=14	–	<5	–	<7	>3

**Table 2:** Recommended color scheme for the BCC Defect Analysis (BDA).

Structure	RGB	CMYK
Perfect bcc crystal	(179,179,179)	#B3B3B3
Surface	(255,255,255)	#FFFFFF
Mono- and di-vacancy, vacancy row	(0,109,179)	#006DB3
Twin boundary, screw dislocation	(255,170,51)	#FFAA33
{110} planar fault	(0,255,0)	#00FF00
(Non-screw) Dislocation	(4,20,168)	#0414A8
Unidentified defect	(223,133,255)	#DF85FF
